# Unravelling the genetic causes of multiple malformation syndromes: A whole exome sequencing study of the Cypriot population

**DOI:** 10.1371/journal.pone.0253562

**Published:** 2021-07-29

**Authors:** Evie Kritioti, Athina Theodosiou, Thibaud Parpaite, Angelos Alexandrou, Nayia Nicolaou, Ioannis Papaevripidou, Nina Séjourné, Bertrand Coste, Violetta Christophidou-Anastasiadou, George A. Tanteles, Carolina Sismani

**Affiliations:** 1 Department of Cytogenetics and Genomics, The Cyprus Institute of Neurology and Genetics, Nicosia, Cyprus; 2 Clinical Genetics Clinic, The Cyprus Institute of Neurology and Genetics, Nicosia, Cyprus; 3 The Cyprus School of Molecular Medicine, The Cyprus Institute of Neurology and Genetics, Nicosia, Cyprus; 4 Aix Marseille Université, CNRS, LNC-UMR 7291, Marseille, France; 5 Clinical Genetics Clinic, The Cyprus Institute of Neurology and Genetics and Archbishop Makarios III Medical Centre, Nicosia, Cyprus; University of Adelaide, AUSTRALIA

## Abstract

Multiple malformation syndromes (MMS) belong to a group of genetic disorders characterised by neurodevelopmental anomalies and congenital malformations. Here we explore for the first time the genetic aetiology of MMS using whole-exome sequencing (WES) in undiagnosed patients from the Greek-Cypriot population after prior extensive diagnostics workup including karyotype and array-CGH. A total of 100 individuals (37 affected), from 32 families were recruited and family-based WES was applied to detect causative single-nucleotide variants (SNVs) and indels. A genetic diagnosis was reported for 16 MMS patients (43.2%), with 10/17 (58.8%) of the findings being novel. All autosomal dominant findings occurred *de novo*. Functional studies were also performed to elucidate the molecular mechanism relevant to the abnormal phenotypes, in cases where the clinical significance of the findings was unclear. The 17 variants identified in our cohort were located in 14 genes (*PCNT*, *UBE3A*, *KAT6A*, *SPR*, *POMGNT1*, *PIEZO2*, *PXDN*, *KDM6A*, *PHIP*, *HECW2*, *TFAP2A*, *CNOT3*, *AGTPBP1* and *GAMT*). This study has highlighted the efficacy of WES through the high detection rate (43.2%) achieved for a challenging category of undiagnosed patients with MMS compared to other conventional diagnostic testing methods (10–20% for array-CGH and ~3% for G-banding karyotype analysis). As a result, family-based WES could potentially be considered as a first-tier cost effective diagnostic test for patients with MMS that facilitates better patient management, prognosis and offer accurate recurrence risks to the families.

## Introduction

Multiple malformation syndromes (MMS) are a heterogeneous group of genetic disorders that cause developmental anomalies in two or more systems and comprise multiple congenital malformations [1]. Some examples of MMS are Rett syndrome, Pitt-Hopkins syndrome and Sjögren-Larsson syndrome, which are primarily characterised by intellectual disability (ID), developmental delay (DD) and congenital anomalies involving different systems such as skeletal anomalies, cardiac malformations and other neurological defects. According to the European surveillance of congenital anomalies (EUROCAT) the 2000–2009 birth prevalence of congenital malformations associated with chromosomal or single gene disorders is 4 in 1000 [2].

Despite the low prevalence of single gene disorders, such rare diseases pose a high public-health concern due to their high morbidity and mortality, healthcare costs and other medical challenges. An accurate diagnosis is therefore vital for better management, prognosis and implementation of potential therapies. A molecular diagnosis can also provide information on reproductive options, recurrence risks, offer genetic counselling, prenatal diagnosis and genetic testing to the family members of the affected patients [3].

The first-tier investigations for MMS include detailed clinical evaluation, biochemical analyses, imaging and genetic conventional diagnostic procedures such as chromosome G-banding, fluorescent *in-situ* hybridisation (FISH), array-comparative genomic hybridisation (CGH) and targeted gene testing. Nevertheless, despite the extensive diagnostic workup that most MMS patients undergo, almost half of them remain undiagnosed [4], a fact that may be accounted to a number of factors, including the low diagnostic potential of conventional methods, applied to this group of patients. In fact, several studies regarding patients with multiple congenital anomalies and/or ID have set the diagnostic yield of array-CGH to be at 10–20% [5,6], while for G-banding chromosomal analysis the diagnostic rate of was ~3%.

The past few decades have seen major progress in the development of diagnostic techniques used to characterise rare genetic diseases, especially with the introduction of next generation sequencing (NGS). Through its unbiased sequencing approach, whole exome sequencing (WES) has unveiled a detection rate of approximately 30% in the molecular diagnosis of rare Mendelian disorders [7,8] and has led to the high discovery rate of novel disease-causing genes [9]. Such discoveries facilitate the elucidation of the mode of inheritance that a Mendelian disease follows and enrich our understanding of the biological mechanisms and functions of these genes, which ultimately opens up the avenue for translational research into new diagnostic and therapeutic approaches.

Family-based WES, where patients with a suspected Mendelian disorder are analysed alongside their healthy parents, has emerged as a rapid and cost-effective approach in the identification of the causal variant [8]. Trio-WES previously demonstrated to successfully reduce the number of candidate variants by 10 times [7] and increased the diagnostic yield compared to sequencing the individual proband alone by excluding a large number of non-pathogenic heterozygous rare variants that would otherwise be flagged as potentially disease-causing [8].

Large cohort studies within Europe, such as the Deciphering Developmental Disorders (DDD) study, performing WES on thousands of patients with developmental anomalies have successfully provided a molecular diagnosis to their patients, including single nucleotide variants (SNVs) and copy number variants (CNVs), and have even discovered novel disease-causing genes [7]. To maximize the chance of finding highly penetrant monogenic causes, this study focuses on patients presenting with more than one congenital manifestation and at least one neurodevelopmental anomaly that may be presented as an early onset severe phenotype.

To our knowledge, the present study is the first evaluation of WES as a tool for investigating undiagnosed MMS patients of the Greek-Cypriot population. Novel findings of family-based WES are described and the introduction of WES to the diagnostic practice in Cyprus is outlined as a tier to accelerate the elucidation of the molecular basis of MMS for each family and ending the odyssey of undiagnosed MMS patients.

## Materials and methods

### Patients

A total of 32 unrelated families with 37 affected individuals were recruited in the study (102 total individual tests) between August 2017 and June 2019. Patients were referred by Clinical Geneticists following careful evaluation to fulfil the study’s criteria.

The inclusion criteria were: (i) presence of congenital malformations or dysmorphic features and at least one neurodevelopmental anomaly; (ii) a prior negative diagnostic workup (including chromosomal G-banding analysis and array-CGH, at a resolution of at least 200kb and/or targeted gene testing and/or Fragile-X syndrome testing; (iii) negative history of exposure to known causative environmental factors including exposure to teratogens, viral infections and alcohol during pregnancy; (iv) availability of detailed phenotypic description including the patient’s family pedigree and any family medical history if applicable; (v) availability of parental samples (vi) signed informed consent by the patient and parents/other family members (if applicable).

This study was approved by the Cyprus National Bioethics Committee under the title “Genetic and Molecular Characterisation of Greek-Cypriot Patients with Multiple Malformation Syndromes (MMS) of Unknown Aetiology” (EEBK/EII2017/19).

### Whole exome sequencing

Genomic DNA (gDNA) was extracted from peripheral blood samples using the QIAmp DNA Blood Midi Kit (Qiagen, Toronto, ON, Canada) and DNA exome library preparation was performed using the TruSeq DNA Exome library preparation kit (Illumina, San Diego, CA, USA) according to the manufacturer’s protocol guidelines. The high-output flow cell and the Illumina NextSeq500 platform were used for paired-end (75x2) sequencing of twelve exomes per run.

### Bioinformatics analysis

NGS analysis was performed using an updated version of an in-house developed pipeline [10] according to the Genome Analysis Toolkit (GATK) Best Practices (version 3.3), executed on the Cy-Tera high performance computer (HPC) clusters of the Cyprus Institute (http://web.cytera.cyi.ac.cy/). Raw reads (FASTQ format) were aligned to the human reference genome (GRCh37/hg19) using the Burrows-Wheeler Aligner (BWA-MEM) software [11]. Variants were called using the GATK HaplotypeCaller and GATK Hardfiltering was performed to remove low confidence calls. Variants were annotated for their effect using the standalone script of the Variant Effect Predictor (VEP) tool (Ensembl release 81) (https://asia.ensembl.org/info/docs/tools/vep/) and further annotated and prioritised using GEMINI (Genome MINIng, version 0.20.1) [12]. In-house filtering guidelines were implemented to isolate variants with a strong correlation to the phenotype. Variants with potential damaging effects were prioritised using *in silico* prediction tools, such as CADD (https://cadd.gs.washington.edu/) as well as PolyPhen (version 2.2.2) (http://genetics.bwh.harvard.edu/pph2/) and SIFT (version 5.2.2) (https://sift.bii.a-star.edu.sg/) for missense variants. Prioritisation was performed according to the suspected mode of inheritance of the disease and minor allele frequencies (MAF) of <1% and <5%, from publicly available databases such as gnomAD exomes r2.0.1 (https://gnomad.broadinstitute.org/) and 1000 Genomes (Phase 3) (http://phase3browser.1000genomes.org/index.html). Filtered variants were inspected with an in-house allele frequency dataset specific to the Greek-Cypriot population containing 166 exomes. Databases such as OMIM (https://omim.org/) and ClinVar (https://www.ncbi.nlm.nih.gov/clinvar/) aided in the identification of known pathogenic variants or established genes contributing to known clinical phenotypes. Individual variants were further manually assessed using publicly available databases and tools such as the public version of the Human Gene Mutation Database (HGMD^®^) (version 2017.4) (http://www.hgmd.cf.ac.uk/ac/index.php) and Mutation Taster (http://www.mutationtaster.org/) respectively. Pathogenicity verdict of variants according to ACMG was assisted with Varsome [13].

*Peddy* tool [14] was used after each run to derive an accurate prediction of the individual’s sex and to check relatedness between samples from the same family/trio. The latter feature is essential when discovering *de novo* variants.

To prevent false-positive results originating from the NGS analysis and variant filtering, the integrative genomics viewer (IGV) [15] was used to establish sufficient coverage of the area enclosing the candidate variant, GC content and presence of variants within low complexity regions.

### Detection of CNVs from WES using ExomeDepth

CNV analysis was carried out using WES data of patients from our cohort who did not have a significant finding (i.e. SNV/indel) that could explain their phenotype.

CNV detection was performed using ExomeDepth (v. 1.1.15) [16]. For this analysis an in-house R script was implemented using R version 3.6 on the Cytera cluster of the Cyprus Institute (http://web.cytera.cyi.ac.cy/).

The analysis was performed with standard parameters using bam files created previously for SNV and indel variant calling procedure as described before. For each reference set, 5–10 samples were used depending on the available unrelated samples in the batch. For potential CNV calls on X chromosome only samples from the same sex were correlated.

A typical experiment produced on average 238 CNV calls. Results were filtered using Bayes factor (BF) >50 [17] and each call was further annotated using the online VEP tool (Ensembl release 81) and BioMart (http://grch37.ensembl.org/biomart/martview/).

### Confirmation and interpretation of candidate variants

All flagged potentially pathogenic variants were initially evaluated in an interdisciplinary manner between the Geneticists, Bioinformaticians and Clinical Geneticists.

Polymerase chain reaction (PCR) amplification and subsequent bi-directional Sanger sequencing using the ABI 3130xl Genetic Analyzer (Applied Biosystems, Foster City, CA, USA) were used to confirm these variants. All primers were designed using the Primer3 web tool (version 0.4.0) (http://bioinfo.ut.ee/primer3-0.4.0/). A list comprising all primers used for the amplification of all candidate variants within this study is included in S1 Table.

All candidate sequence variants were classified as pathogenic, likely pathogenic, variants of uncertain significance (VUS), likely benign and benign (Table 1), using the standards and guidelines of the American College of Medical Genetics and Genomics (ACMG) [18], assisted by the automatic classification offered by Varsome [13]. All potential causative sequence variants discovered from this study were deposited in ClinVar database (https://www.ncbi.nlm.nih.gov/clinvar) with accession numbers SCV001443796—SCV001443812.

**Table 1:** Clinical and molecular findings of molecularly characterised MMS patients.

Patient	Gender	Age at WES	Main clinical characteristics	Gene	Exon	Nucleotide change	Protein change	Coding impact	Inheritance	Zygosity	Novel/Known	MAF (gnomAD)	CADD score	Variant classification^a^	OMIM Diagnosis
**1**	F	8	Microcephaly, microsomia, dwarfism, mild-moderate ID, mild-moderate DD, FTT	*PCNT*	14	NM_006031.6: c.2407C>T	p.Gln803*	Nonsense	AR	Homozygous	Novel	N/A	40	Pathogenic (PVS1, PM2, PP3, PP4)	Microcephalic osteodysplastic primordial dwarfism, type II (OMIM #210720)—AR
**2**	M	4	GDD, hypotonia, glossoptosis, tapering fingers, plagiocephaly, over-folded ear helices, myopia	*UBE3A*	12	NM_130839.4: c.2410C>T	p. Gln804*	Nonsense	*De novo*	Heterozygous	Novel	N/A	45	Pathogenic (PVS1, PS2, PM2, PP3, PP4)	Angelman Syndrome (OMIM #105830)—AD
**3**	F	7	GDD, FTT, static leukoencephalopathy, microcephaly, pinched nose, bifid first molar, unilateral blepharoptosis	*KAT6A*	16–17	NM_006766.5: c.3039+1G>A	N/A	Splice donor defect	*De novo*	Heterozygous	Novel	N/A	25.1	Pathogenic (PVS1, PS2, PM2, PP3, PP4)	Arboleda-Tham syndrome (OMIM #616268)—AD
**4**	F	59	Moderate-severe ID, cataplexy, obesity, tapering fingers, drowsiness, speech delay	*SPR*	3	NM_003124.5: c.655C>T	p.Arg219*	Nonsense	AR	Homozygous	rs779204655 [19]	3.182x10^-5^	36	Pathogenic (PVS1, PM2, PP4, PP5)	Dystonia, dopa-responsive, due to sepiapterin reductase deficiency (OMIM #612716)—AR
**5**	F	48	Moderate-severe ID, cataplexy, obesity, tapering fingers, drowsiness, speech delay, rigidity, Parkinsonism	*SPR*	3	NM_003124.5: c.655C>T	p.Arg219*	Nonsense	AR	Homozygous	rs779204655 [19]	3.182x10^-5^	36	Pathogenic (PVS1, PM2, PP4, PP5)	Dystonia, dopa-responsive, due to sepiapterin reductase deficiency (OMIM #612716)—AR
**6**	M	22	Severe ID, DD, congenital muscular dystrophy, absent speech, sensorineural hearing loss, hyperCkemia, stereotypies, severe visual impairment, cataract, hyperactivity, behavioral abnormalities, macular dystrophy	*POMGNT1*	4	NM_001243766.1: c.304G>T	p.Glu102*	Nonsense	AR	Compound heterozygous	Novel	N/A	36	Pathogenic (PVS1, PM2, PP3, PP4)	Muscular dystrophy-dystroglycanopathy (congenital with brain and eye anomalies), type A, 3 (OMIM #253280)—AR
				*POMGNT1*	5	NM_001243766.1: c.385C>T	p.Arg129Trp	Missense			rs375431575 [20,21]	7.961x10^-6^	27.6	Likely pathogenic (PM1, PM2, PM3, PP2, PP3, PP4)	
**7**	M	18	Severe ID, DD, congenital muscular dystrophy, absent speech, sensorineural hearing loss, hyperCkemia, stereotypies, severe visual impairment, hydrocephaly, eczematous rash, squint	*POMGNT1*	4	NM_001243766.1: c.304G>T	p.Glu102*	Nonsense	AR	Compound heterozygous	Novel	N/A	36	Pathogenic (PVS1, PM2, PP3, PP4)	Muscular dystrophy-dystroglycanopathy (congenital with brain and eye anomalies), type A, 3 (OMIM #253280)—AR
				*POMGNT1*	5	NM_001243766.1: c.385C>T	p.Arg129Trp	Missense			rs375431575 [20,21]	7.961x10^-6^	27.6	Likely pathogenic (PM1, PM2, PM3, PP2, PP3, PP4)	
**8**	M	26	Severe ID, DD, seizures, primary microcephaly, tetraparesis, spasticity with permanent contractures, long fingers, thickened alveolar ridge, thickened gums, strabismus, scoliosis, exaggerated reflexes, bilateral foot deformities	*PIEZO2*	24	NM_022068.3: c.3655G>A	p.Val1219Met	Missense	AR	Compound heterozygous	rs73946020	2.229x10^-3^	22	VUS (PS3, PS4, PM2, PM3, PP4, BP4, BP6)	Arthrogryposis, distal, with impaired proprioception and touch (OMIM #617146)—AR
				*PIEZO2*	32	NM_022068.3: c.4724G>A	p.Arg1575Gln	Missense			rs374051556	4.04x10^-5^	24	VUS (PS3, PS4, PM2, PP4, BP4, BP6)	
**9**	M	26	Bilateral iris defects, extensive anterior synechiae, posterior embryotoxon, nystagmus, glaucoma, cataracts, bilateral hydronephrosis, vesicoureteric obstruction, bilateral megaureter and bipolar disorder	*PXDN*	16	NM_012293.3: c.2098G>T	p.Gly700*	Nonsense	AR	Homozygous	Novel	N/A	41	Pathogenic (PVS1, PM2, PP3, PP4)	Anterior segment dysgenesis 7, with sclerocornea (OMIM #269400)—AR
**10**	M	27	Severe ID, FTT, microcephaly, spasticity, large prominent teeth, dysplastic ears, sparse eyebrows, high arched palate, short stature, frequent otitis media, dry skin, broad thumbs, sparse teeth, hypoplastic teeth	*KDM6A*	12	NM_021140.3: c.1192C>T	p.Gln398*	Nonsense	*De novo*	Heterozygous	Novel	N/A	37	Pathogenic (PVS1, PM2, PP3, PP4)	Kabuki syndrome (OMIM #300867)—AD
**11**	M	25	DD, mild myopathy, right orchiopexy, horseshoe kidney, anger issues, shirt terminal phalanges, lactose intolerance, lipomastia, slanting palpebral fissures, brachydactyly, tapering fingers, mild retrognathia, increased laxity, coordination issues	*PHIP*	31	NM_017934.7: c.3631_3634delCAAA	p.Gln1211Aspfs*13	Frameshift	De novo	Heterozygous	Novel	N/A	N/A	Pathogenic (PVS1, PM2, PP3, PP4)	Chung-Jansen syndrome (OMIM #617991)—AD
**12**	F	22	Severe ID, DD, self-injurous behaviour, absent speech, hypotonia, contractures of upper limbs, hand flapping, scoliosis, macrostomia, broad nasal tip, squint, irregular menses, facial dysmorphic features	*HECW2*	20	NM_020760.4: c.3542C>G	p.Ala1181Gly	Missense	*De novo*	Heterozygous	Novel	N/A	24.9	Likely pathogenic (PS2, PM2, PP3, PP4)	Neurodevelopmental disorder with hypotonia, seizures, and absent language (OMIM #617268)—AD
				*HECW2*	20	NM_020760.4: c.3587A>G	p.Lys1196Arg	Missense	*De novo*	Heterozygous	Novel	N/A	27.5	Pathogenic (PS2, PM1, PM2, PP3, PP4)	
**13**	M	3	GDD, ID, camptodactyly, upslanting palpebral fissures, microstomia, coloboma, sparse hair	*TFAP2A*	4	NM_003220.2: c.763A>G	p.Arg255Gly	Missense	*De novo*	Heterozygous	rs121909574 [22–24]	N/A	24	Pathogenic (PS2, PM1, PM2, PP2, PP3, PP4, PP5)	Branchiooculofacial syndrome (OMIM #113620)—AD
**14**	M	9	GDD, ID, speech delay, short stature, craniocynostosis, strabismus, anal artesia,hypospadias, small chin, 5^th^ finger clinodactyly, dysplastic kidneys, scoliosis	*CNOT3*	8	NM_014516.4: c.520G>A	p.Glu174Lys	Missense	*De novo*	Heterozygous	rs1238165628	3.99x10^-6^	33	Likely pathogenic (PS2, PM2, PP2, PP3)	Intellectual developmental disorder with speech delay, autism, and dysmorphic facies (OMIM #618672)—AD
**15**	F	Deceased	GDD, ID, FTT, cerebellar atrophy, microcephaly, tremors, chilblains, erythroderma, feeding difficulties, hypotonia, seizures, spinal muscular atrophy, death in childhood	*AGTPBP1*	10	NM_015239.2: c.820_821delCA	p.Gln274GlufsTer17	Frameshift	AR	Homozygous	Novel	N/A	N/A	Pathogenic (PVS1, PM2, PP3, PP4)	Neurodegeneration, childhood-onset, with cerebellar atrophy (OMIM #618276)—AR
**16**	F	44	Severe ID, absent speech, epileptic encephalopathy, coarse facial features, asymmetric skull, macroglossia, hand biting, stereotypies	*GAMT*	2	NM_138924.3: c.327G>A	p.Lys109 =	Splice donor defect/Synonymous	AR	Homozygous	rs80338735 [25–30]	1.86x10^-4^	14.7	Pathogenic (PVS1, PM2, PP4, PP5, BP4)	Cerebral creatine deficiency syndrome 2 (OMIM #612736)—AR

Abbreviations: F—Female; M—Male; ID—Intellectual Disability; DD—Developmental Delay; FTT—Failure to Thrive; GDD—Global Developmental Delay; AR—Autosomal Recessive; AD—Autosomal Dominant.

^a^ Variant classification according to the current ACMG guidelines.

In the case of a confirmed molecular diagnosis the family was informed and was offered genetic counselling accordingly.

### Additional investigations/functional studies

#### Splice site variant analysis

For splice site variants with an unclear significance, Human Splicing Finder (http://www.umd.be/HSF/index.html) was used to predict the variants’ effect on splicing. In cases with a predicted positive effect, RNA was extracted from Epstein-Barr Virus (EBV) transformed lymphoblastoid cell lines using the RNeasy midi kit (Qiagen) and underwent reverse transcription using the ProtoScript^®^ First Strand cDNA Synthesis Kit (New England Biolabs, Ipswich, MA, USA). PCR amplifications were performed using custom designed primers (S2 Table) and Sanger sequencing was performed using the ABI 3130xl Genetic Analyzer (Applied Biosystems).

#### *PIEZO2* constructs, site-directed mutagenesis and transformations

Mutations Val1219Met and Arg1575Glu were introduced in full-length human *PIEZO2* cDNA by site-directed mutagenesis using Phusion^®^ High-Fidelity DNA Polymerase (New England Biolabs) and primers Piezo2-V1219M-pcDNA-F, Piezo2-V1219M-pcDNA-R, Piezo2-R1575Q-pcDNA-F and Piezo2-R1575Q-pcDNA-R (S3 Table) according to a modified version of the QuickChange II XL Site-Directed Mutagenesis Kit (Agilent) (conditions are available upon request). The primer pair for the mutagenesis of Arg1575Glu was designed manually as to contain the altered base mid-sequence and the primer pair for Val1219Met was designed using the QuickChange^®^ Primer Design Program (Agilent) [31]. The mutated constructs were transformed in DH5 alpha turbo competent *E*. *coli* cells. Plasmid cDNA was purified using the Qiagen Plasmid Maxi kit according to the manufacturer’s protocol. Sanger sequencing was used to confirm the successful mutagenesis of both variants followed by sequential digestion using *ApaI* and *NotI*.

#### Cell culture and transient transfection

PIEZO1-deficient HEK293T cells (Human embryonic kidney) [32] were grown in Dulbecco’s Modified Eagle Medium containing 4.5 mg.mL^-1^ glucose, 10% (vol/vol) FBS, 100 U.mL^-1^ (1%) penicillin/streptomycin. Cells were plated onto poly-D-lysine-coated 12-mm round glass coverslips (Corning) in 24-well plates and transfected using lipofectamine 2000 (Invitrogen) according to the manufacturer’s instruction. Each well was transfected with 600 ng.mL^-1^ of specified constructs. For double mutant experiments, cells were co-transfected with WT + Arg1575Glu constructs or Val1219Met + Arg1575Glu constructs at a ratio of 1:2 (300 ng.mL^-1^+ 600 ng.mL^-1^ plasmid per condition). All transfections were supplemented with 300 ng.mL^-1^ of GFP plasmid to visualise transfected cells and GFP positive cells were recorded 24–48 hours later.

#### Electrophysiology

Patch-clamp experiments were performed in standard whole-cell recordings using an Axopatch 200B amplifier (Axon Instruments). Patch pipettes had a resistance of 1.5–2.5 MΩ when filled with an internal solution consisting of (in mM) 140 CsCl, 10 Hepes, 5 EGTA, 1 CaCl_2_, 1 MgCl_2_, 4 MgATP, and 0.4 Na_2_GTP (pH adjusted to 7.3 with CsOH). The extracellular solution consisted of (in mM) 133 NaCl, 3 KCl, 1 MgCl_2_, 10 Hepes, 2.5 CaCl_2_, 10 glucose (pH adjusted to 7.3 with NaOH). All experiments were done at room temperature. Currents were sampled at 20 kHz and filtered at 2 kHz. Leak currents before mechanical stimulations were subtracted off-line from the current traces. Clampfit 10.7 (Molecular Devices) software was used to analyse recordings and biophysical parameters. PRISM 7.0 (GraphPad) software was used for statistical analysis (non-parametric Krustal-Wallis test). All chemicals were sourced from Sigma-Aldrich.

#### Mechanical stimulation

Mechanical stimulation was achieved using a firepolished glass pipette (tip diameter 3–4 μm) positioned at an angle of 80° to the cell being recorded. Downward movement of the probe toward the cell was driven by a Clampex controlled piezo-electric crystal microstage (E625 LVPZT Controller/Amplifier, Physik Instrumente). The probe had a velocity of 0.7 μm.ms^-1^ during the ramp segment of the command for forward motion, and the stimulus was applied for 150 ms. A series of mechanical steps in 0.5 μm increments was applied every 10 s. Inward MA currents were recorded at a holding potential of -80 mV.

## Results

### Patients

**Fig 1:**
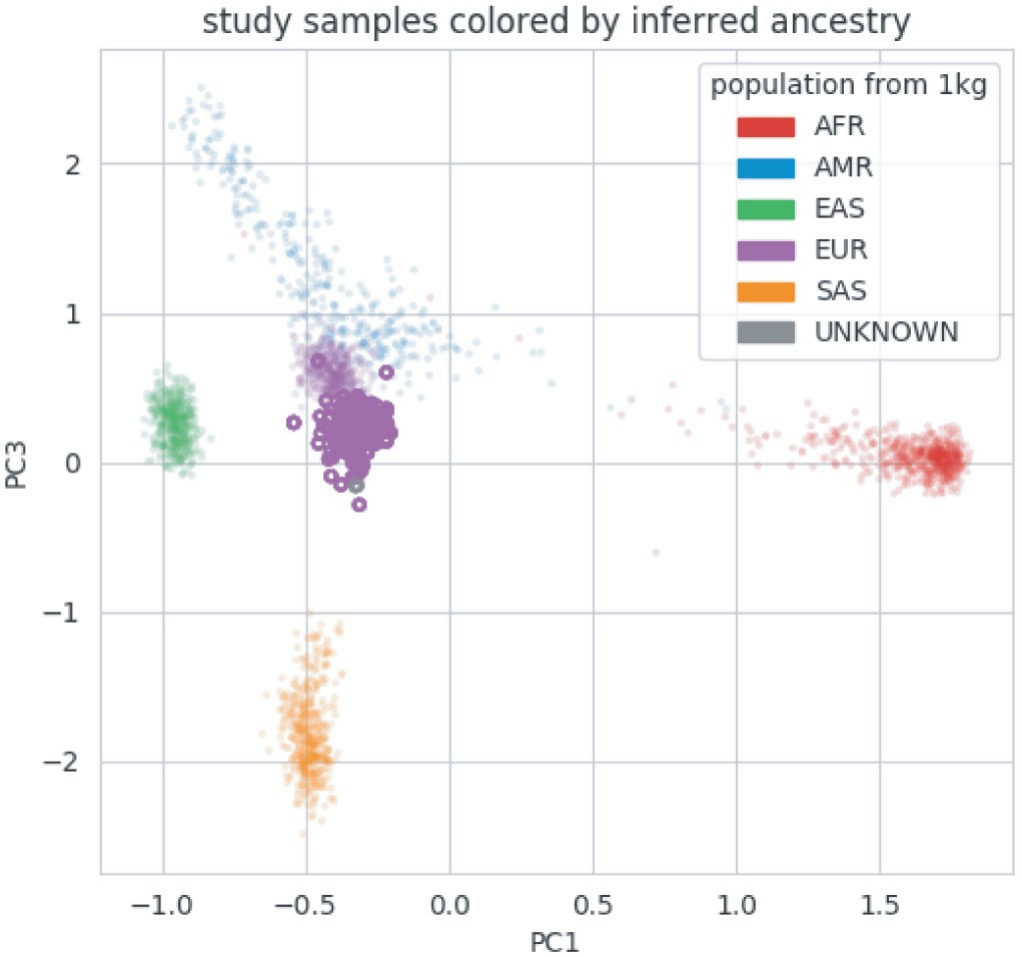
Principal Component Analysis (PCA) using *peddy* tool, of samples (*n =* 166) from in-house dataset (in purple) projected to those from 1000 genomes. The samples of this study are included in the dataset.

Thirty-two unrelated families with 37 patients met the inclusion criteria of this study as outlined in the Methods section. The patient group included 16 children (43.2%) and 21 adults (56.7%). The mean age of patients at the time of referral for WES was 21 (ranging from 3.5 to 59), with a nearly equal male to female ratio (18:19). The vast majority of the individuals included within this cohort are from the European population and more specifically from Greek-Cypriot origin. The observed ancestry composition of our cohort was estimated with a randomized Principal Component Analysis (PCA) as shown in Fig 1.

The most common clinical characteristics within our cohort were ID and DD. A wide spectrum of congenital anomalies, dysmorphic features and other neurological deficits were presented including short stature, microcephaly and seizures respectively.

Three (9.4%) of the recruited families were self-reported consanguineous. All parents included in the cohort were apparently healthy.

### Whole exome sequencing

#### Coverage/Depth

Based on the 100 samples that underwent WES within this cohort, the mean target coverage was 59X, with 89% of the target bases achieving at least 10X coverage.

### Molecular results

#### Detection rate

Family-based WES and subsequent analysis performed on 32 families revealed 17 potentially pathogenic SNVs or small indels in 16 MMS patients, achieving an overall detection rate of 43.2% (16/37). All molecular diagnoses and clinical information are summarised in Table 1. The clinical information of the recruited MMS patients without a molecular diagnosis is available upon request.

### Candidate variants

All 17 candidate variants had a strong correlation with the patient’s phenotype (Table 1).

Based on the distribution of the confirmed candidate variants, missense (7/17, 41.1%) were most abundant, followed closely by nonsense (6/17, 35.3%) variants. For the detection of potentially causative variants, *in silico* tools such as PolyPhen2 and SIFT predicted pathogenicity for all 7 flagged missense mutations and most mutations had a CADD score of more than 20, suggesting deleteriousness (Table 1). Based on the ACMG guidelines for the classification of sequence variants, the majority of our findings were classified as pathogenic (11/17, 64.7%). Based on our in-house dataset, the flagged variants detected in the 16 MMS patients were not identified in any other individuals referred to the lab. An autosomal recessive mode of inheritance was the most common between the diagnosed MMS patients (9/16, 56.3%). All candidate variants were identified in genes known to cause Mendelian monogenic disorders with the majority of the variants being described as novel (10/17, 58.8%), meaning they have not been reported before in the literature or in publicly available databases. An analytical summary of these statistics is illustrated in Table 2.

**Table 2:** Distribution of Mendelian mode of inheritance, novelty of variants and variant type of index cases with a molecular diagnosis.

	**Number of diagnosed patients (percentage %)**
**Mode of inheritance**	
**Dominant**	**7 (43.8%)**
Autosomal dominant	6 (37.5%)
X-linked dominant	1 (6.3%)
**Recessive**	**9 (56.3%)**
Homozygous	6 (37.5%)
Compound heterozygous	3 (18.8%)
X-linked recessive	0
	**Number of candidate pathogenic variants (percentage %)**
**Variant novelty**	
Known	7 (41.2%)
Novel	10 (58.8%)
**Type of mutation**	
Nonsense	6 (35.3%)
Missense	7 (41.2%)
Frameshift	2 (11.8%)
Splice defect	2 (11.8%)

All 7 autosomal dominant conditions resulted from *de novo* mutations, whereas autosomal recessive conditions resulted from homozygous and compound heterozygous mutations. All patients with *de novo* disease-causative variants were born to apparently healthy, non-consanguineous parents.

Patient 12 had 2 heterozygous *de novo* variants in gene *HECW2* causing neurodevelopmental disorder with hypotonia, seizures, and absent language (OMIM #617268). The variants were not present in neither of the parents upon Sanger confirmation and when observing our NGS data the two variants were located on the same reads (S2 Fig).

Of the recessive diagnosed disorders, variant p.Arg219* observed in *SPR* (patients 4 and 5) has been previously identified in patients with similar phenotypes [19]. Although some of the other variants including compound heterozygous variants p.Glu102* and p.Arg129Trp found in *POMGNT1* (patients 6 and 7) and p.Val1219Met and p.Arg1575Gln identified in *PIEZO2* (patient 8) are present in databases such as ClinVar and gnomAD (Table 1), they have not been phenotypically described in the literature.

Of the conditions that were caused by monogenic homozygous mutations, one was found in *PCNT* in patient 1, from a quartet (two affected siblings and both parents) with consanguineous parents. Another was identified in *PXDN* (patient 9) within a trio with parents who were third cousins and the other four cases were found in *SPR* (patients 4 and 5), *AGTPBP1* (patient 15) and *GAMT* (patient 16), as mentioned above, within different trios with apparently unrelated parents.

All of the above variants following a recessive mode of inheritance demonstrated clear segregation from apparently unaffected carrier parents to their affected offspring.

Collectively, 15 genes (*PCNT*, *UBE3A*, *KAT6A*, *SPR*, *POMGNT1*, *PIEZO2*, *PXDN*, *KDM6A*, *PHIP*, *HECW2*, *TFAP2A*, *CNOT3*, *AGTPBP1* and *GAMT*) with presumed pathogenic SNVs were identified within this cohort.

### CNV findings from WES data

Upon CNV analysis of all patients with a negative result from family-based WES, no significant CNVs were identified that could explain the patients’ clinical presentations.

### Further experimental investigations

**Fig 2:**
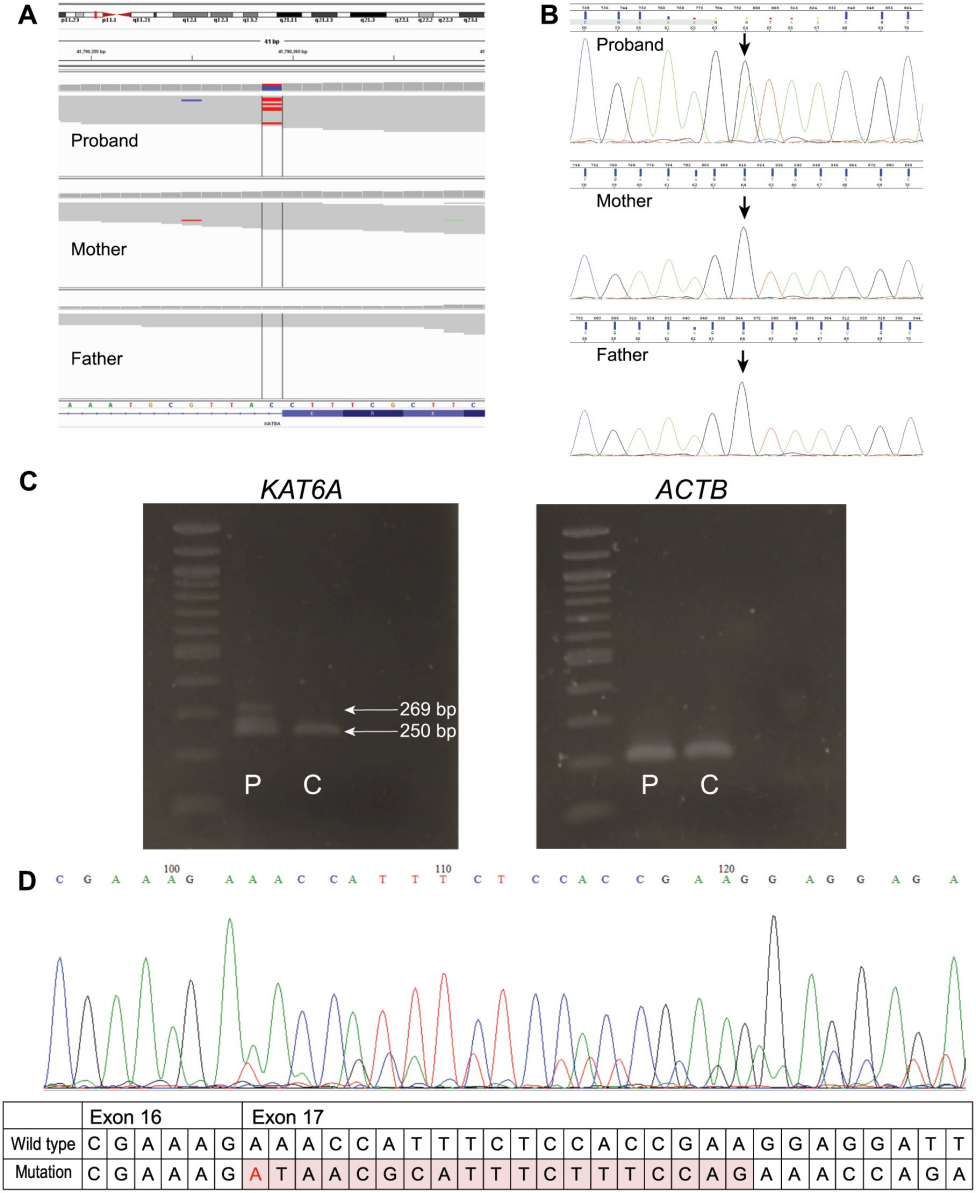
Analysis of the c.3039+1G>A splice variant. A—IGV image illustrating presence of de novo splice variant c.(3039+1G>A) in *KAT6A*, identified in patient 3 and absent in parents. B—Electropherograms showing presence of c.(3039+1G>A) in DNA of proband (G/A) and absence in both parents (G/G) C—RT-PCR of *KAT6A* and actin (*ACTB*) cDNA transcripts of patient 3 (P) and a healthy control (C). D—The functional effect of c.(3039+1G>A) splice variant in patient 3, which was identified by sequencing the cDNA product from RNA extracted from patient’s lymphoblastoid cell lines. The splice donor variant resulted in the intron retention of 19bp and a frameshift change.

A predicted pathogenic splice site variant c.(3039+1G>A) in *KAT6A* was identified in patient 3. Sanger sequencing of cDNA derived from patient lymphoblastoid cell lines revealed that the functional effect of the splice variant resulted in a 19bp intron retention at the donor site of exon 16 (Fig 2), which is predicted to result in the protein’s truncation.

**Fig 3:**
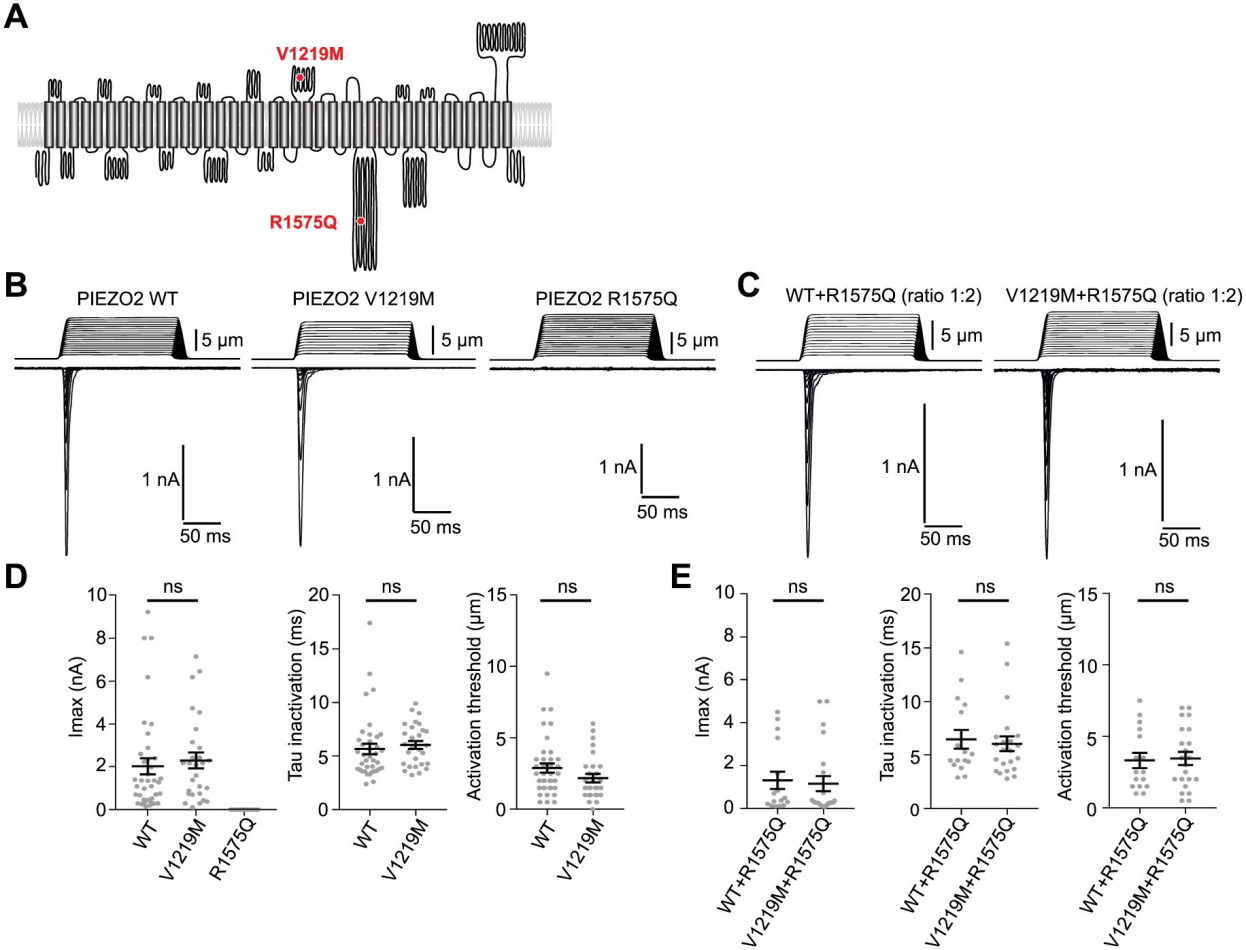
**A**—Schematic topology of PIEZO2 protein with position of the two identified mutation. **B, C**—Typical example of mechanically-activated currents recorded in HEK293T-P1KO cells transiently transfected with each specified constructs (600ng expression plasmid per condition) (B) or co-transfected with WT+R1575Q or V1219M+R1575Q at a ratio of 1:2 (300ng+600ng plasmid per condition) (C). Holding potential is -80mV. Top traces represent the displacement of the stimulation probe, bottom traces represent the recorded currents. Traces corresponding to mechanical stimulations incremented by 0.5 μm are superimposed. **D, E**—Average maximal current amplitude (left panel), inactivation time-constant (middle panel) and threshold for mechanical current activation (right panel). Cells were recorded 24 to 48 hours post transfection transfection (n = 38, 28 and 14 for WT, V1219M and R1575Q, respectively; n = 16 and 22 for WT+R1575Q and V1219M+R1575Q, respectively; mean±s.e.m.; Mann-Whitney test).

For patient 8, two missense variants were identified in a compound heterozygous form in gene *PIEZO2*. PIEZO2 homo-trimers form mechanically activated (MA) ion channels involved in touch and proprioception [33–36], whose mutations, either gain- or loss-of-function, have been linked to MMS with overlapping clinical phenotypes [37]. We generated human *PIEZO2* constructs with p.(Val1219Met) maternally inherited mutation and p.(Arg1575Gln) paternally inherited mutation to test functional consequences of these mutations on PIEZO2 channel activity. Heterologous expression of these mutants was done in HEK-P1 KO cells to avoid contamination by endogenous MA channels and compared to WT channels (Fig 3). Expression of Val1219Met mutant induces MA currents similar to WT PIEZO2 regarding current amplitude, inactivation kinetics and activation threshold. Interestingly, expression of Arg1575Gln mutant does not induce any detectable MA channel activity (Fig 3B and 3D).

Since Val1219Met mutation seems to have no effect on properties of recorded MA currents, we tested if its association with Arg1575Gln mutant could induce MA channels with altered functional properties. However, co-expression of Val1219Met and Arg1575Gln mutants induced MA currents with similar characteristic than co-expression of WT and Arg1575Gln mutant (Fig 3C and 3E), arguing that Val1219Met associated to Arg1575Gln mutation do not induce MA channels with altered biophysical properties. Altogether, these experiments show that heterologous expression of Val1219Met mutant induces MA currents similar to WT currents, and that Arg1575Gln mutation is deleterious and induces loss-of-function.

## Discussion

Here we present the first genomic investigation performed on MMS patients from the Greek-Cypriot population. This study highlights the high detection rate of 43.2% obtained by WES for a challenging group of undiagnosed patients, identifying a total of 17 potentially causative variants.

A range of inheritance patterns, types of mutations and a high number of novel findings were identified (Table 2). Fifty-three percent of the total pathogenic variants involved autosomal recessive disorders, which demonstrates the high potential of family-based WES in diagnosing recessive diseases in patients who have already undergone an extensive diagnostic workup. The number of detected autosomal recessive disorders is significantly higher compared to other similar studies accounting for 27% and 34.3% in patient groups of 416 and 2000 respectively [38,39]. This increase in recessive diseases detected within the Cypriot population may be accounted to the small population size of Cyprus as well as the small sample size of this study. Due to the high prevalence of founder effect mutations observed in regional studies, especially those with small population sizes, the study’s group will resume the study in order to identify the true frequency of potential founder effect mutations within the Cypriot population. Specifically, a high frequency (>0.05) of a founder effect mutation related to β-thalassemia has already been extensively demonstrated within the Cypriot population in the past [40]. Concurrently, the lower number of *de novo* variants detected may account for their novelty and for the fact that they may be present in genes that have not been linked to a disease before, stressing the importance of depositing novel molecular findings to freely-accessible databases. Doing so will subsequently increase knowledge within the scientific community and accelerate diagnosis for other MMS patients. We also observed that missense mutations were abundant in our cohort (43.2%) as it has also been observed in a recent study on patients with neurodevelopmental disorders and multiple congenital anomalies (40.8%) [41]. Even though the frequencies of autosomal recessive disorders and missense variants detected in this study are higher or similar to other larger studies, a more reliable frequency can be achieved with the continuity of this study and subsequent increase of the study’s sample size.

Literature regarding rare genetic syndromes is limited, therefore even in the cases where already known disease-causing variants are identified, they offer valuable additions to limited pre-existing knowledge. In our study, the identification of already known disease-causing variants has helped expand the spectrum of phenotypes. More specifically, of the 7 known mutations identified within our cohort only three have been characterised both molecularly and phenotypically in the past and are present in OMIM. These mutations were detected in genes *SPR*, *TFAP2A* and *GAMT* [19,22,23,25–30]. The current study contributed to the phenotypic spectrum of the disorder caused by mutations found within *SPR*, especially since the affected siblings with a variant p.(Arg219*) in *SPR* are significantly older than the majority of previously reported cases. Obesity and a Prader-Willi-like manifestation are present in both of our *SPR* patients, both features being novel to patients with sepiapterin reductase deficiency (SRD). Such findings can ultimately offer a new and faster avenue for the clinical diagnosis of younger SRD patients.

Novel phenotypes were also identified in patient 15 who is a carrier of a homozygous *AGTPBP1* variant p.(Gln274GlufsTer17), which is a novel variant within a known disease-associated gene. The patient’s clinical manifestations included novel phenotypes such as erythroderma and an abnormal metabolic profile, which have not been reported before in patients with recessive mutations in *AGTPBP1*.

Known variants with a strong genotype-phenotype correlation within well-established disease-causing genes were easily identified as the primary flagged variants. Such findings include two compound heterozygous variants in *POMGNT1* in two male siblings (patients 6 and 7), with typical MRI findings of patients with *POMGNT1* mutations (S1 Fig), a homozygous variant in *PCNT* in patient 1 who was subsequently diagnosed with microcephalic osteodysplastic primordial dwarfism, type II (MOPDII) and patient 2 with a variant in *UBE3A* causative for Angelman syndrome. Although the latter two are novel variants, other flagged variants such as ones identified within *SPR* and *TFAP2A* have already been characterised in the literature before, making the diagnoses of these patients easier and marking the end of the diagnostic odyssey of these families much faster. For variants found within genes that have recently been linked to a genetic disorder and for variants with uncertain significance, such as ones located within *KAT6A* and *PIEZO2*, further work was carried out to assess their pathogenicity.

Even though RNA sequencing can demonstrate the direct effect of a mutation on the RNA or even protein level, WES provided insight to investigate the effect of splice variants at an RNA-level without the need to perform RNA sequencing. This was more specifically observed in patient 3, with a splice donor variant in *KAT6A* (c.3039+1G>A) that led to an intron retention of 19bp.

*PIEZO2* mutations in humans lead to disease of variable clinical phenotypes with overlapping clinical features including arthrogryposis syndrome [37]. The two previously reported missense variants identified in *PIEZO2* from patient 8 were tested functionally. The paternally inherited variant p.(Arg1575Gln) was functionally inactive, as its heterologous expression induced no detectable MA channel activity. Arg1575Gln is located within a sequence connecting Beam and Latch domains of the protein whose alteration could affect gating of Piezo2 channels [42,43] or could induce mis-fold of the protein leading to its degradation. No functional difference was identified in MA currents induced by the maternally inherited variant p.(Val1219Met) whose mutation lies in the TM21-TM22 extracellular loop [43]. Since PIEZO channels are formed by homo-trimeric assembly, co-expression experiments were done with deleterious p.(Arg1575Gln) in excess. Co-expression of p.(Arg1575Gln) with WT induces MA currents similar to WT currents suggesting no dominant negative effect. Co-expression of both mutants induces MA currents similar to WT or p.(Val1219Met) currents, excluding an interaction-dependent mechanism between mutants. It cannot be ruled out that the p.(Val1219Met) mutation may have effects on channel activity that are not detected due to sensitivity limitations of our functional assay. Another hypothesis is that p.(Val1219Met) mutation could alter PIEZO properties that are not related to its ion channel activity or that are dependent from the cellular context, such as interaction with partners absent in heterologous expression system. Nevertheless, patient 8 clinical phenotype is reminiscent of the various clinical phenotypes associated with biallelic loss-of-function or dominant gain-of-function *PIEZO2* mutations [44–46] suggesting that biallelic p.(Val1219Met) and p.(Arg1575Gln) mutations alter *PIEZO2* function leading to MMS, which could therefore be causative for the patient’s phenotype.

It has been shown that approximately 2% of all *de novo* SNVs are multinucleotide variants (MNVs) occuring 20bp-5kb from one another [47,48] and constitute an important, sometimes undetected cause of genetic disorders. Based on a recent study on 6688 trio-WES cases, *de novo* MNVs were more frequently observed within genes associated with developmental disorders and were suggested to be more damaging than SNVs, even when the MNVs were both missense mutations [49]. This appears to be the case of patient 12, where two missense variants 45bp apart were identified in *HECW2*. Both variants were classified as *likely pathogenic* and their combination appears to be deleterious. This class of SNV variants is very challenging and special attention is required for their interpretation and detection.

Since the use of WES can give different detection rates for different patient groups, this study was also able to highlight the importance of patient selection and setting strict criteria in achieving a high detection rate (43.2%). Receiving a detailed phenotypic description from the clinicians and having stricter patient inclusion criteria can ultimately lead to higher detection rates being achieved using WES.

Despite a high detection rate, the limitations of WES as a diagnostic tool are associated to its incomplete coverage of exonic regions, insufficient sequencing of areas with high GC content, low complexity regions, stretches of repeats, inability in detecting mosaicism in the parents due to the limited depth of coverage of the technique and the challenges in detecting CNVs.

CNVs not detected by array-CGH or WES are still left undiscovered, which could account for a large number of cases that are left undiagnosed. Recent advances in CNV detection using WES data have proven to be beneficial and have increased the overall diagnostic rate of various studies [50,51]. Based on the literature, the diagnostic rates of CNV detection on patients using WES data vary widely among different tools [16]. In this cohort, all patients had already undergone an extensive diagnostic workup until the time of CNV analysis, leaving little room for the detection of pathogenic CNVs.

In order to increase the rate of accurate genetic diagnosis for MMS patients, future advances in higher read depths, the use of whole genome sequencing (WGS), long-read sequencing and reanalysis of WES data could potentially make it possible to accurately identify pathogenic SNVs and CNVs in a single experiment.

## Conclusions

This is the first genomic study on MMS patients from the Greek-Cypriot population. WES provided a potential genetic diagnosis for 43.2% of a cohort of MMS patients, with a previous negative diagnostic workup, and included the discovery of rare variants, most of which are novel. The high diagnostic yield and efficacy of WES observed in this study suggest that WES could even be considered as a first-tier cost effective diagnostic test over conventional diagnostic approaches for MMS patients when a specific disorder is not suspected. The high detection rate indicates that WES is a valuable tool for the efficient diagnosis of patients with MMS and highlights the importance of selecting patients that have more than one congenital anomaly and/or neurodevelopmental disorder or an early onset severe phenotype. Lastly, we propose that the genes in which flagged variants were identified in this study could be added in clinical exome sequencing (CES) and related panels for MMS. For the undiagnosed cases, WES data reanalysis and WGS will be carried out in the near future.

## Supporting information

S1 Fig Example of strong genotype-phenotype correlation illustrated through MRI findings of patients 6 and 7 showing hydrocephalus and vermian hypoplasia; typical characteristics of patients with mutations in *POMGNT1*. (TIF)

S2 Fig IGV image illustrating presence of *de novo* variants p.(Ala1181Gly) and p.(Lys1196Arg) in *HECW2*, in patient 13, on same reads. (TIF)

S1 Table List of primer pairs used in Sanger sequencing for validation of flagged variants. The synthesis scale and shipping condition of all primers were 0.02 μmol and diss. 100 μM respectively. [F: forward; R: reverse]
(DOCX)

S2 Table Primers used for cDNA sequencing for confirmation of functional effect of splice change in patient 3. (DOCX)

S3 Table Primers used for site-directed mutagenesis in human *PIEZO2* pcDNA3.1. (DOCX)

S1 Raw images (PDF)
