# Supplementary figures and images for: Unravelling the genetic causes of multiple malformation syndromes: A whole exome sequencing study of the Cypriot population
Source: PLoS One. 2021 Jul 29;16(7):e0253562. doi: 10.1371/journal.pone.0253562 (PMC8320927; doi:10.1371/journal.pone.0253562)

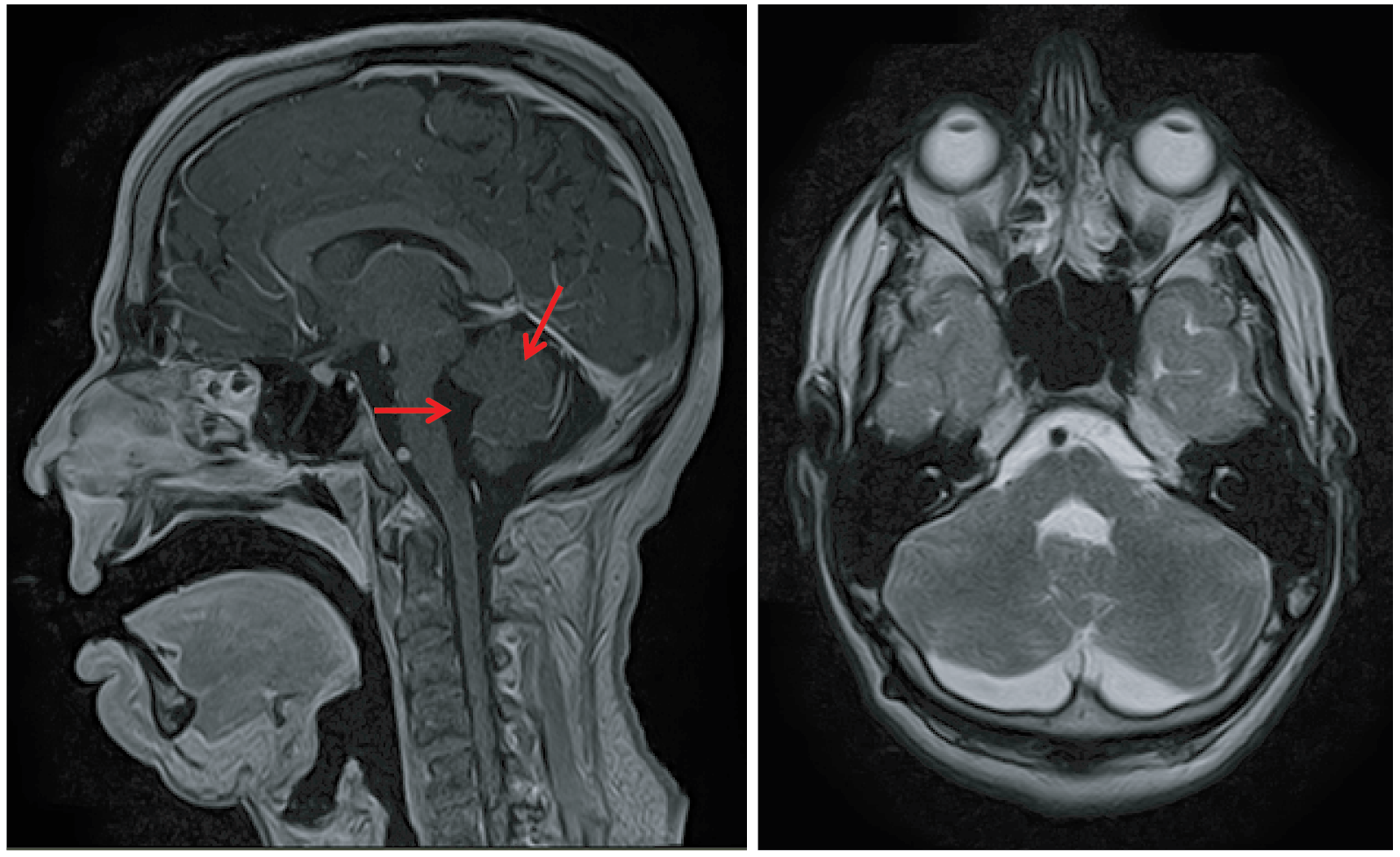

Supplement: S1 Fig — (TIF) [file pone.0253562.s001.tif]

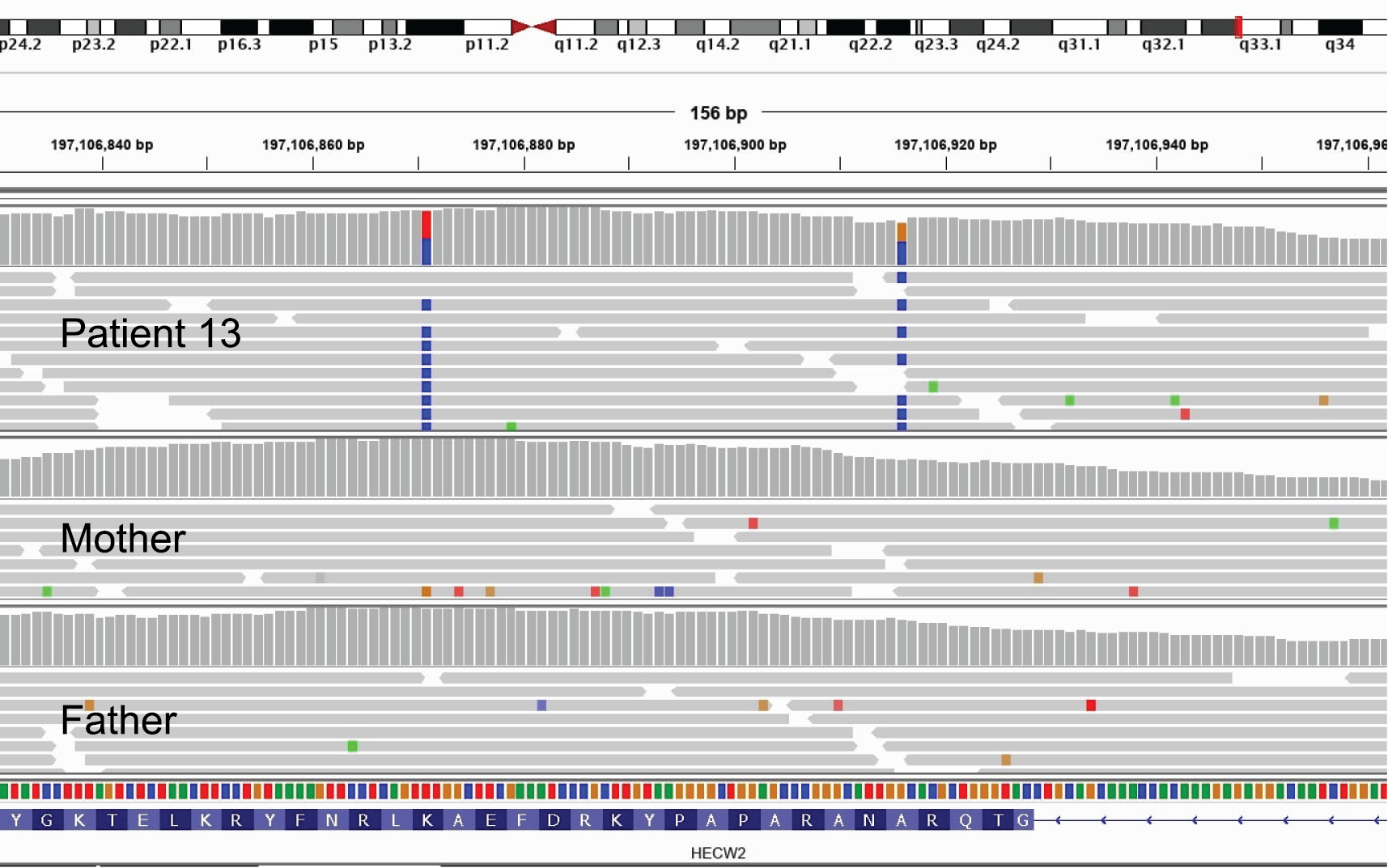

Supplement: S2 Fig — (TIF) [file pone.0253562.s002.tif]

*ACTB*

100bp  
DNA Ladder

Proband

Control

*KAT6A*

Proband

Control

← 269 bp  
← 250 bp

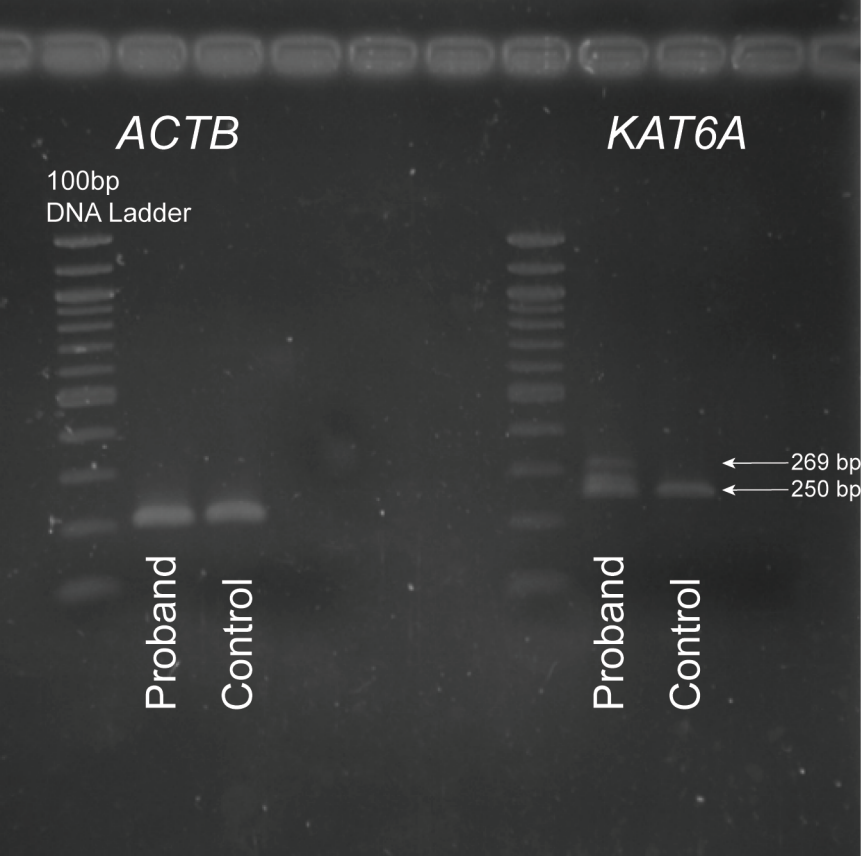

Supplement: S1 Raw images — (PDF) [file pone.0253562.s006.pdf]
