# Supplementary material for: Unravelling the genetic causes of multiple malformation syndromes: A whole exome sequencing study of the Cypriot population
Source: PLoS One. 2021 Jul 29;16(7):e0253562. doi: 10.1371/journal.pone.0253562 (PMC8320927; doi:10.1371/journal.pone.0253562)
Supplement: S1 Table — The synthesis scale and shipping condition of all primers were 0.02 μmol and diss. 100 μM respectively. [F: forward; R: reverse] (DOCX) [file pone.0253562.s003.docx]

| **Primer Name** | **Amplicon coordinates (hg19/GRCh37)** | **Sequence (5’-3’)** | **Length (bp)** | **Tm (°C)** | **GC%** | **Self Compl.** | **Self 3’ Compl.** |
| --- | --- | --- | --- | --- | --- | --- | --- |
| PCNT-A-F | chr21:47783569-47783588 | AAGGCTGAATCCGAGAAACA | 20 | 60 | 45 | 3 | 2 |
| PCNT-A-R | chr21:47783663-47783682 | CAAGGACCTCTCCAGATCCA | 20 | 60 | 55 | 5 | 2 |
| UBE3A-F | chr15:25585132-25585152 | TCACGAATGTGCTCAGAAACT | 21 | 59 | 43 | 4 | 1 |
| UBE3A-R | chr15:25585505-25585528 | TTGGAAATTTTTAAATCACCAGAA | 24 | 59 | 25 | 8 | 2 |
| KAT6A-F | chr8:41798094-41798113 | GTTTGTTTGCATCCCAGGAG | 20 | 61 | 50 | 4 | 2 |
| KAT6A-R | chr8:41798428-41798446 | CAGTGAGAGCAGCGAGGAG | 19 | 60 | 63 | 3 | 0 |
| SPR-F | chr2:73118351-73118370 | GCCAAGATGACGTGTTTCCT | 20 | 60 | 50 | 4 | 0 |
| SPR-R | chr2:73118664-73118683 | AAGCCAAAAACATGGGCTTA | 20 | 60 | 40 | 5 | 2 |
| POMGNT1-R129W-F | chr1:46661239-46661258 | ATGAGGGTGGGGACTCTGT | 19 | 59 | 58 | 3 | 1 |
| POMGNT1-R129W-R | chr1:46661834-46661853 | GCAGGAGGTGATGTTTCAAG | 20 | 58 | 50 | 3 | 1 |
| POMGNT1-E102*-F | chr1:46662316-46662336 | TTCCCACTTAGGCAGTAGACC | 21 | 58 | 52 | 3 | 0 |
| POMGNT1-E102*-R | chr1:46662652-46662671 | ATGAAGACCCAGAGCCAGAG | 20 | 59 | 55 | 2 | 0 |
| PIEZO2-R1575Q-F | chr18:10736427-10736446 | CTGGGAGCAGGCATCATTAC | 20 | 61 | 55 | 3 | 1 |
| PIEZO2-R1575Q-R | chr18:10736800-10736821 | CGTTGTGGTTCTCTTATGGACA | 22 | 60 | 45 | 3 | 2 |
| PIEZO2-V1219M-F | chr18:10759241-10759260 | TGGGGGAAGTGAAATTATGC | 20 | 60 | 45 | 4 | 2 |
| PIEZO2-V1219M-R | chr18:10759619-10759640 | GGGAGAAGAGTGGTGAAGAAGA | 22 | 60 | 50 | 1 | 0 |
| COL27A1-F | chr9:116982014-116982033 | CACCCTGAAGGTGACAACAC | 20 | 59 | 55 | 7 | 2 |
| COL27A1-R | chr9:116982145-116982164 | CAGAATCTCGCTCCTGTTCC | 20 | 60 | 55 | 5 | 0 |
| PXDN-F | chr2:1657292-1657311 | GTGGGAAAGAAAATGCTGGA | 20 | 60 | 45 | 2 | 1 |
| PXDN-R | chr2:1657496-1657515 | TCCGAGGGATCCTTACACAG | 20 | 60 | 55 | 6 | 1 |
| KDM6A-F | chrX:44918597-44918617 | TCTCTATGAATCCTGCAACCA | 21 | 58 | 43 | 4 | 1 |
| KDM6A-R | chrX:44918759-44918778 | CCCTCCTTCTGCCTGAGTGT | 20 | 62 | 60 | 3 | 0 |
| PHIP-F | chr6:79671353-79671372 | GGTGGAAAGATCACCAGCTT | 20 | 59 | 50 | 4 | 2 |
| PHIP-R | chr6:79671507-79671526 | TATTGCCTCAGCATTTGTGG | 20 | 60 | 45 | 3 | 2 |
| HECW2-F | chr2:197106732-197106751 | CAATTTCCTAGAGGCCCAAC | 20 | 59 | 50 | 4 | 0 |
| HECW2-R | chr2:197107112-197107131 | ACCAAAATGCCACAAAGAGG | 20 | 60 | 45 | 3 | 1 |
| TFAP2A-F | chr6:10404588-10404607 | TTGGCTCTACGCTCTTCTCC | 20 | 60 | 55 | 2 | 0 |
| TFAP2A-R | chr6:10404942-10404961 | CTGTCCAAGTCCAACAGCAA | 20 | 60 | 50 | 4 | 0 |
| CNOT3-F | chr19:54649150-54649169 | GCTAAGATTGGTCCCCACAG | 20 | 60 | 55 | 3 | 1 |
| CNOT3-R | chr19:54649420-54649439 | CGAGGATGGAGTCATTGTCC | 20 | 60 | 55 | 3 | 1 |
| AGTPBP1-F | chr9:88272161-88272180 | TGACCATTTCCTCCAACTCA | 20 | 59 | 45 | 3 | 1 |
| AGTPBP1-R | chr9:88272540-88272559 | GAAACAAATGCCAGGAGAGC | 20 | 60 | 50 | 3 | 2 |
| GAMT-F | chr19:1399497-1399516 | CTGTGATACGTCCCCTCACC | 20 | 60 | 60 | 4 | 1 |
| GAMT-R | chr19:1399842-1399861 | TTGGATCATCGAGTGCAATG | 20 | 61 | 45 | 5 | 3 |
