# Supplementary material for: Unravelling the genetic causes of multiple malformation syndromes: A whole exome sequencing study of the Cypriot population
Source: PLoS One. 2021 Jul 29;16(7):e0253562. doi: 10.1371/journal.pone.0253562 (PMC8320927; doi:10.1371/journal.pone.0253562)
Supplement: S2 Table — (DOCX) [file pone.0253562.s004.docx]

| **Primer name** | **Sequence** |
| --- | --- |
| KAT6A-cDNA-F | CGCTACAGTGAGGGTGACAG |
| KAT6A-cDNA-R | CGGAGTCAGAATCTTCAAAAGG |
